# Gastrotomy

**Published:** 1890-08-23

**Authors:** 


### SURGERY.

### GASTROTOMY.

Mr. Mayo Robson, surgeon to the Leeds Infirmary, has reported a case of gastrotomy performed for cancerous disease of the œsophagus. The subject had suffered for five months from difficulty of taking solid food, which regurgitated or was vomited. For some time before admission liquids were also rejected. Bougies were arrested at a point 12½ inches from the teeth, and not even the smallest could be passed through the stricture. For several days before the operation he was fed by enemata. Antiseptic precautions were first taken, the abdomen being purified by a carbolic acid dressing worn for twelve hours. A vertical incision for three inches was made from below the costal margin along the outer border of the left rectus. The parietal peritoneum was then excised and sutured to the skin margin. The stomach was then fixed to the abdominal wall by silk sutures, the previous insertion of two loops of silver wire serving to mark the spot where the stomach was to be opened, and to manipulate the stomach while the silk sutures were being applied. The wound was then dressed with sal alembroth gauze. On the fourth day afterwards, a small opening was made into the stomach with a tenotomy knife, through which food was introduced. Ten days afterwards the patient was allowed to sit up. Eventually a short celluloid tube with a stopper was introduced, which the patient could take out and reintroduce, and through which he could feed himself. Two months afterwards he had gained a stone and a half in weight. His improved condition continued for six months, when his general health began to fail, and he died from exhaustion, due to the gradual progress of the original malignant disease of the œsophagus. But he lived in a stage of comparative comfort for eleven months, during the whole of which time he never took a particle of food excepting through the artificial opening into the stomach. Although gastrotomy for non-malignant obstruction is more successful, when performed for cancer of the œsophagus, it is rarely so successful as in Mr. Mayo Robson's case.

